# Effects of the dual peroxisome proliferator-activated receptor-α/γ agonist aleglitazar on renal function in patients with stage 3 chronic kidney disease and type 2 diabetes: a Phase IIb, randomized study

**DOI:** 10.1186/1471-2369-15-180

**Published:** 2014-11-18

**Authors:** Luis Ruilope, Markolf Hanefeld, A Michael Lincoff, Giancarlo Viberti, Sylvie Meyer-Reigner, Nadejda Mudie, Dominika Wieczorek Kirk, Klas Malmberg, Matthias Herz

**Affiliations:** Hospital 12 de Octubre, Clinical Science, Madrid, Spain; Study Centre Prof. Hanefeld, GWT-TUD GmbH, Dresden, Germany; Cardiovascular Medicine, Cleveland Clinic, Ohio, USA; Cardiovascular Division, King’s College London School of Medicine, London, UK; F. Hoffmann-La Roche, Basel, Switzerland; Department of Cardiology, Institution of Medicine, Karolinska Institutet, Stockholm, Sweden

**Keywords:** Aleglitazar, Pioglitazone, PPAR-α/γ, AleNephro, eGFR, Serum creatinine, Type 2 diabetes

## Abstract

**Background:**

Type 2 diabetes is a major risk factor for chronic kidney disease, which substantially increases the risk of cardiovascular disease mortality. This Phase IIb safety study (AleNephro) in patients with stage 3 chronic kidney disease and type 2 diabetes, evaluated the renal effects of aleglitazar, a balanced peroxisome proliferator-activated receptor-α/γ agonist.

**Methods:**

Patients were randomized to 52 weeks’ double-blind treatment with aleglitazar 150 μg/day (n = 150) or pioglitazone 45 mg/day (n = 152), followed by an 8-week off-treatment period. The primary endpoint was non-inferiority for the difference between aleglitazar and pioglitazone in percentage change in estimated glomerular filtration rate from baseline to end of follow-up. Secondary endpoints included change from baseline in estimated glomerular filtration rate and lipid profiles at end of treatment.

**Results:**

Mean estimated glomerular filtration rate change from baseline to end of follow-up was –2.7% (95% confidence interval: –7.7, 2.4) with aleglitazar versus –3.4% (95% confidence interval: –8.5, 1.7) with pioglitazone, establishing non-inferiority (0.77%; 95% confidence interval: –4.5, 6.0). Aleglitazar was associated with a 15% decrease in estimated glomerular filtration rate versus 5.4% with pioglitazone at end of treatment, which plateaued to 8 weeks and was not progressive. Superior improvements in high-density lipoprotein cholesterol, low-density lipoprotein cholesterol and triglycerides, with similar effects on glycosylated hemoglobin were observed with aleglitazar versus pioglitazone. No major safety concerns were identified.

**Conclusions:**

The primary endpoint in AleNephro was met, indicating that in stage 3 chronic kidney disease patients with type 2 diabetes, the decrease in estimated glomerular filtration rate after 52 weeks’ treatment with aleglitazar followed by 8 weeks off-treatment was reversible and comparable (non-inferior) to pioglitazone.

**Trial registration:**

NCT01043029 January 5, 2010.

**Electronic supplementary material:**

The online version of this article (doi:10.1186/1471-2369-15-180) contains supplementary material, which is available to authorized users.

## Background

Type 2 diabetes (T2D) increases the risk of cardiovascular (CV) disease and microvascular complications, including diabetic nephropathy [1]. Multiple CV risk factors—including hypertension, dyslipidemia, insulin resistance and vascular inflammation–drive vascular risk in patients with T2D, necessitating comprehensive management strategies [2–4]. A multifactorial approach to treatment of T2D, including lifestyle intervention, control of glycosylated hemoglobin (HbA1c), low-density lipoprotein cholesterol, blood pressure (including renin–angiotensin–aldosterone system [RAAS] inhibition), insulin resistance and low-grade inflammation, significantly reduces CV events, but considerable CV risk remains [5, 6].

Aleglitazar is designed to work through balanced activation of peroxisome proliferator-activated receptors (PPARs)-α and -γ to improve insulin sensitivity, dyslipidemia and inflammation [7–9]. In the SYNCHRONY study [9], a daily dose of 150 μg aleglitazar over 16 weeks significantly improved HbA1c, fasting plasma glucose (FPG) and the lipid profile, whilst ameliorating inflammatory markers in patients with T2D and normal renal function. Aleglitazar at this dose was well tolerated, with a similar incidence of adverse events compared with placebo [9]. However, a non-progressive, dose-related increase in serum creatinine (SCr) was observed during aleglitazar treatment, with a corresponding decrease in estimated glomerular filtration rate (eGFR) [9]. The significance of these effects for kidney function was investigated further in a dedicated renal function study (SESTA R) [10], which evaluated the effects of a supratherapeutic dose of aleglitazar (600 μg/day for 6 months) on measured GFR (mGFR) and eGFR in patients with T2D and normal or mildly impaired renal function (eGFR Modification of Diet in Renal Disease [eGFR_MDRD_] 60 to 120 mL/min/1.73 m^2^). SESTA-R established that mean percentage changes in eGFR correlated with true mGFR, and confirmed that the effect of aleglitazar therapy on SCr and GFR was non-progressive and reversible upon treatment discontinuation.

Data from SYNCHRONY and SESTA-R suggest good short-term safety of aleglitazar in patients with normal or mildly impaired kidney function and also provide evidence for beneficial effects of aleglitazar on multiple markers of CV risk, namely hyperglycemia, diabetic dyslipidemia, insulin resistance and inflammation [11]. To assess the longer-term safety and efficacy of aleglitazar in patients with T2D, additional trials were initiated, including a renal safety study (AleNephro [NCT01043029]) and a CV outcomes trial in patients with T2D following an acute coronary syndrome (ACS; AleCardio [NCT01042769]) [12]. Although aleglitazar’s development was recently halted due to lack of efficacy in CV outcomes indicating no CV benefit, and PPAR-related class side effects in the post-ACS T2D population [12], the renal effects of dual PPAR-α/γ activation remain of interest—in particular, the development of another PPAR-α/γ agonist, tesaglitazar—was terminated over concerns about the degree and potential lack of reversibility of elevations in SCr.

Here, we present the results of the Phase IIb AleNephro study, which was designed to evaluate the renal effects of aleglitazar treatment in patients with T2D and more advanced kidney impairment (stage 3 chronic kidney disease [CKD]) using the PPAR-γ agonist pioglitazone as active comparator over 52 weeks. Reversibility of renal effects was also assessed via an 8-week treatment-free follow-up period.

## Methods

### Study design

AleNephro was a randomized, double-blind, active-controlled, parallel-group, multicenter Phase IIb renal function non-inferiority safety study. Following a 2-week screening period, patients received a once-daily dose of 150 μg aleglitazar or 45 mg pioglitazone (Takeda Pharmaceutical Company, Osaka, Japan) for 52 weeks, added to pre-existing antihyperglycemic therapy and/or diet and exercise. After termination of treatment, patients were followed for 8 weeks, with visits in Weeks 4 and 8 to evaluate reversibility of renal effects.

### Study population

Patients were recruited between May 27, 2010 and May 13, 2011. Enrolment was carried out by the principal investigator or designee at participating clinical sites. Inclusion criteria were age ≥ 18 years, diagnosis of T2D and moderately impaired kidney function (CKD stage 3, as defined by eGFR_MDRD_ ≥ 30 and < 60 mL/min/1.73 m^2^), plus the following at screening: naïve to the use of oral antihyperglycemic agents or on monotherapy or combination therapy with no more than two antihyperglycemic medications; HbA1c 6.5–10%; FPG ≤ 13.3 mmol/L; urinary albumin/creatinine ratio (UACR) ≤ 3000 μg/mg; body mass index from 25.0 kg/m^2^ (Asian patients: from 23.0 kg/m^2^) to 35.0 kg/m^2^.

Exclusion criteria were: known diagnosis of renal disease (except diabetic nephropathy), congestive heart failure New York Heart Association class II to IV, known macular edema or impaired liver function (alanine aminotransferase or aspartate aminotransferase > 3 times the upper limit of normal). Patients were also excluded if they were currently on, or had previously received, the following treatments: thiazolidinedione or insulin (with the exception of emergency cases, in which insulin was given for < 7 consecutive days), or medications interfering with measurement of creatinine (e.g. cimetidine, trimethoprim, probenecid, sulfonamides, procaine or thiazolesulfone); treatment with fibrates in the 3 months preceding the screening visit; chronic therapy with a non-steroidal anti-inflammatory drug (with the exception of prophylactic stable low-dose aspirin) 1 month prior to screening; or changes in antihypertensive therapy in the last 3 months or in statins in the last month before screening or likely to require changes during the study. All individuals provided written informed consent. The study was conducted according to the principles of the Declaration of Helsinki and the laws and regulations of the participating countries. The protocol was approved by independent review committees or institutional review boards. A full list of the ethics committees and institutional review boards that approved the study is included in the supplementary materials (Additional file 1: Table S1).

### Randomization and masking

Patients, study site personnel and sponsor were all blinded to treatment assignment. At the baseline visit, patients were randomly assigned (via an interactive voice-response system) in a 1:1 ratio to receive orally either 150 μg aleglitazar tablets and placebo capsules matching pioglitazone capsules, or 45 mg pioglitazone capsules and placebo tablets matching aleglitazar tablets. The patient randomization numbers were generated by Roche and maintained by an unblinded statistician. The investigator or designee entered the case report form number (CRF; patient number) on the electronic CRF (given to a patient at visit 2 at the time of randomization) and entered the corresponding randomization number for allocation to the treatment groups in the appropriate place on each patient’s eCRF.

The patient randomization numbers were allocated sequentially in the order in which the patients were enrolled according to the specification document agreed with the randomization company (S-Clinica).

The password-protected and/or encrypted electronic master randomization list was kept in a central repository by the Roche Biometrics and Drug Safety Departments. No open key to the code was available at the study center, to the Roche monitors, project statisticians, or to the project team at Roche. Randomization was stratified by eGFR values at screening < 45 versus ≥ 45 mL/min/1.73 m^2^. The dose of 150 μg aleglitazar was selected based on the Phase II dose-ranging study data, which demonstrated improvements in lipid and glycemic parameters with a favorable safety profile [9]. All medications were administered once daily in the morning for 52 weeks, in addition to pre-existing prescribed antihyperglycemic therapy and/or diet and exercise management.

### Study objective and endpoints

The primary objective of the study was to evaluate changes in eGFR following 52 weeks of daily treatment with 150 μg aleglitazar and 8 weeks’ follow-up observation after the last study medication, in comparison with 45 mg pioglitazone. Secondary endpoints included percentage and absolute change from baseline in eGFR and lipid profiles at end of treatment. Tertiary endpoints included change from baseline to end of treatment and after 8 weeks of follow-up in additional renal parameters, and time to first occurrence of any component of the triple-composite renal endpoint (end-stage renal disease [ESRD], confirmed doubling of SCr from baseline [confirmed at least 4 weeks later] or confirmed increase in SCr of 50% [confirmed within 1 week and leading to discontinuation of treatment]), or the double-composite renal endpoint (ESRD or any doubling of SCr from baseline). Safety endpoints included adverse events, clinical laboratory tests, electrocardiography, vital signs, physical examination, peripheral edema, and CV symptoms including events adjudicated by an independent Clinical Events Committee (CEC).

### Procedures

Blood samples were taken at Weeks 2, 4, 8, 12, 16, 20, 26, 39, 52 (treatment period), 56, and 60 (follow-up) to monitor renal, hematologic and biochemical parameters and to assess glycemic control. Blood lipid parameters were measured at baseline and again in Weeks 16, 26, and 52. First morning urine samples were collected at baseline, Weeks 12, 26 and 52, and at both follow-up visits. Three urine samples were collected for each data point: on the morning of the visit and on two preceding mornings. Dipstick urine analysis was used to measure protein, glucose, blood, pH, nitrites, and ketones at alternate visits. Safety and tolerability were assessed throughout the study. All laboratory analyses were conducted at a central laboratory (Covance Central Laboratory Services [Indianapolis, USA; Geneva, Switzerland; and Singapore]). eGFR was calculated using the MDRD equation [13].

### Statistical analysis

The primary analysis was non-inferiority of aleglitazar to pioglitazone evaluated as a difference in percentage change in eGFR_MDRD_ from baseline to end of follow-up (8 weeks after end of treatment). Protocol predefined non-inferiority to pioglitazone was inferred if the lower limit of the two-sided 95% confidence interval (CI) of the difference in eGFR change was greater than –7.5%. The margin of non-inferiority chosen (lower CI of the difference of –7.5%) was considered to be clinically relevant and was agreed by the Food and Drug Administration and European Medical Agency, taking into account the inherent variability in creatinine levels and the anticipated mean natural annual progression in GFR loss in patients with T2D and CKD ranging from 0.9 up to 4 mL/min/1.73 m^2^[14–16].

**Figure 1:**
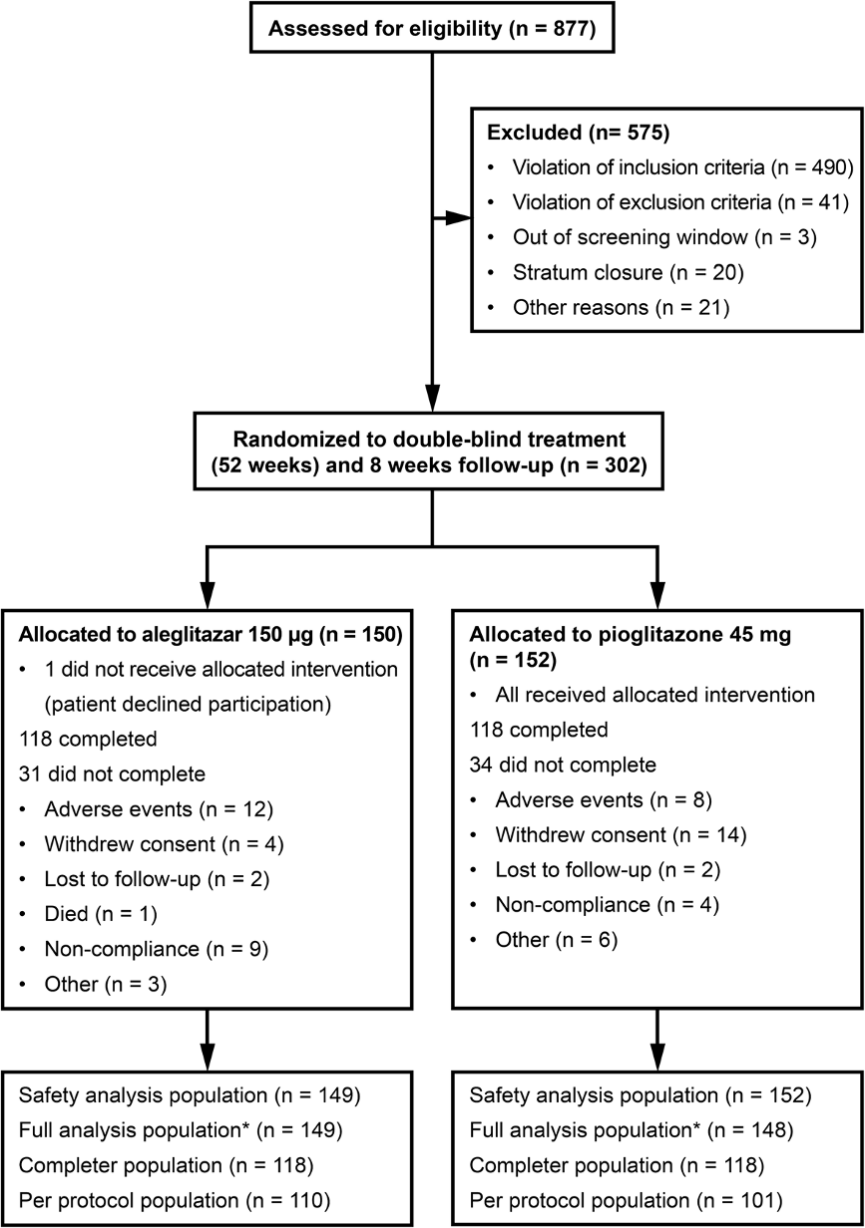
**Patient disposition and study populations.** Safety analysis population: all patients who received at least one dose of the study drug. Full analysis population: all patients who received at least one dose of study drug, and had an evaluable baseline and at least one evaluable post-baseline measurement of serum creatinine. Completer population: all patients included in the full analysis population who completed 52 weeks of double-blind treatment. Per-protocol population: completers without major protocol violations (defined prior database lock and unblinding). * 140 patients on aleglitazar and 132 patients on pioglitazone included in the full analysis population had at least one follow-up measurement of serum creatinine ≥ 21 days after last treatment intake, and therefore were included in the primary analysis.

With a planned sample size of 150 patients enrolled per treatment group (300 patients in total), it was expected that approximately 132 patients (approximately 15% drop-out rate) per group would be included in the full analysis set (FAS) population. This sample size of 132 patients per group provided 80% power for testing the null hypothesis for the primary variable (eGFR), using a one-sided α-level of 0.025. As per protocol, the FAS, following an intent-to-treat principle, consisted of all patients who were randomized, had at least one randomized study drug intake, and had an evaluable baseline and at least one evaluable post-baseline SCr measurement. Imputation using the last-observation-carried-forward method was pre-specified at the study design stage in the protocol as a simple and unambiguous methodology and was hence carried out at the analysis stage. The primary endpoint was assessed by analysis of covariance (ANCOVA) to determine the least squares mean changes from baseline and 95% CI. The ANCOVA model included the percentage change in eGFR between baseline and end of follow-up as the dependent variable and treatment, region, eGFR stratum, UACR stratum (> 300 versus ≤ 300 μg/mg) as fixed effects with a strata interaction term, and baseline eGFR as the covariate. Equivalent ANCOVA models were used to calculate percentage and absolute change for secondary and tertiary endpoints to end of treatment or end of follow-up. P-values are reported and values below 0.05 may be referred as nominally statistically significant. However, no adjustment for multiplicity across secondary and tertiary analyses was done. ANCOVA assumptions were examined and for the endpoints that did not follow normal distribution (UACR, renin, aldosterone, urinary fractional sodium excretion, alpha glutathione S-transferase [α-GST], N-acetyl-β-D-glucosaminidase [NAG] and low-density lipoprotein cholesterol), analysis was on a log-transformed scale with dependent variable expressed as log of relative change from baseline. In these cases, differences were reverse-transformed and results were presented as the percentage change in geometric means. Analogous repeated measures ANCOVA was used for descriptive analyses. Pre-specified subgroup analyses were done using models as above, omitting the interaction term. In order to avoid too few observations in each stratum, models applied to change in UACR albuminuria subgroups were further simplified by omitting fixed terms for region and strata. The protocol pre-specified the last-observation-carried-forward principle to account for missing values of continuous variables. For end of follow-up endpoints, last-observation-carried-forward was only applied where a value was available at least 3 weeks after the last study drug intake. The primary analysis population was the full analysis population, using the intent-to-treat approach (Figure 1). Safety analyses were carried out on the safety population. Continuous variables defined in the protocol as safety were analyzed on the safety population as absolute change from baseline to end of treatment or end of follow-up using ANCOVA model as described for primary endpoint. All analyses were done with SAS (version 9.2) in UNIX.

## Results

### Patient disposition and baseline characteristics

AleNephro was conducted at 62 sites in 13 countries across Europe, South America, Asia and Australia. Of 877 patients screened, 302 patients were randomized to 150 μg/day aleglitazar (n = 150) or 45 mg/day pioglitazone (n = 152) (Figure 1). Of these, 118 in each group completed 52 weeks of the study treatment.

Baseline characteristics were well balanced (Table 1), with patients in both treatment regimens having similar baseline values for eGFR and UACR.

**Table 1:** **Baseline characteristics and medication use (full analysis population)**

Characteristic	Aleglitazar 150 μg (n = 149)	Pioglitazone 45 mg (n = 148)
Patient characteristics*		
Age, years	66. 8 ± 8.0	68.2 ± 7.6
Women, n (%)	74 (50)	78 (53)
Race, white, n (%)	129 (87)	120 (81)
BMI, kg/m^2^	29.8 ± 3.3	29.8 ± 3.1
Median duration of diabetes, years	9.6 (0.4–40.2)	10.2 (0.2–48.2)
Systolic blood pressure, mmHg	130.5 ± 8.3	132.1 ± 10.4
Diastolic blood pressure, mmHg	77.6 ± 7.1	76.6 ± 8.7
Smokers, n (%)	8 (5)	4 (3)
Laboratory data*		
HbA1c_,_ %	7.5 ± 0.9	7.6 ± 0.9
FPG, mmol/L	8.5 ± 2.3	8.7 ± 2.2
Triglycerides, mmol/L	2.3 ± 1.6	2.2 ± 1.2
HDL-C, mmol/L	1.2 ± 0.3	1.2 ± 0.3
LDL-C, mmol/L	2.7 (2.1–3.4)	2.9 (2.3–3.5)
**Markers of renal function**		
eGFR, mL/min/1.73 m^2^	46.5 ± 10.2	46.7 ± 10.2
SCr, μmol/L	123.6 ± 30.5	121.4 ± 29.2
Cystatin-C, μg/mL	1.2 ± 0.4	1.2 ± 0.3
UACR, mg/mmol^†^	2.0 (0.27–257.97)	1.9 (0.24–551.33)
**Markers of RAAS**		
Renin, pmol/L	34.4 (17.8–130.5)	27.0 (17.8–87.0)
Aldosterone, pmol/L	194.2 (111.0–332.9)	194.2 (111.0–305.1)
Sodium excretion (fractional), %	1.5 (1.0–1.9)	1.5 (1.1–2.1)
**Renal tubular markers**		
α-GST, μg/L	3.1 (3.1–3.1)	3.1 (3.1–3.9)
NAG, U/L	2.9 (1.6–4.7)	3.2 (1.8–5.1)
**Baseline medication use, n (%)**		
Glycemic control treatment		
Drug-naïve	6 (4)	7 (5)
Oral antidiabetic agents	143 (96)	141 (95)
Sulfonylurea	112 (75)	112 (76)
Metformin	87 (58)	95 (64)
Antihypertensive medication	138 (93)	140 (95)
Diuretics	73 (49)	81 (55)
ACE inhibitors and/or ARB	120 (81)	121 (82)
Statins	78 (52)	68 (46)
Aspirins^‡§^	65 (44)	64 (43)

ACE = angiotensin-converting enzyme; ARB = angiotensin receptor blocker; BMI = body mass index; eGFR = estimated glomerular filtration rate; HbA1c = glycosylated hemoglobin; HDL-C = high-density lipoprotein cholesterol; LDL-C = low-density lipoprotein cholesterol; RAAS = renin–angiotensin–aldosterone system; SCr = serum creatinine; UACR = urine albumin-to-creatinine ratio.

*Mean ± standard deviation or median (interquartile range) unless otherwise specified. ^†^Minimum–maximum range quoted. ^‡^Aspirin, aspirin/diltiazem, aspirin DL-lysine or salicylic acid. ^§^Total number of patients who received at least one treatment throughout the study.

Although chronic therapy with non-steroidal anti-inflammatory drug was an exclusion criterion, prophylactic stable low dose of aspirin was permitted and aspirin intake during study was recorded in approximately 42% of patients in both treatment groups (Table 1).

### Statistical analysis

Of the 302 patients enrolled into the study, 150 patients were allocated to the aleglitazar group and 152 patients to the pioglitazone group. Of these, four patients in the pioglitazone group were excluded from the FAS because of no evaluable post-baseline SCr measurement (n = 148), and one patient in the aleglitazar group did not receive any study medication, and so was also excluded (n = 149). Additionally, 25 patients did not have evaluable SCr measurements at least 3 weeks after last study drug intake (n = 9 on aleglitazar and n = 16 on pioglitazone). Therefore, 140 patients in the aleglitazar group and 132 in the pioglitazone group were included in the primary analysis, providing at least 80% power for the comparison.

### Renal parameters

Mean percentage changes from baseline to end of treatment and end of follow-up in renal function and renin–angiotensin–aldosterone system (RAAS) markers, including eGFR, SCr, cystatin-C, renin, aldosterone, urinary fractional sodium excretion, α-GST and NAG, are summarized in Table 2.

**Table 2:** **Change in renal function and RAAS markers from baseline to end of treatment and follow-up**

Parameter, LS mean percentage change (95% CI)	EOT		EOF	
	Aleglitazar	Pioglitazone	Aleglitazar	Pioglitazone
	150 μg	45 mg	150 μg	45 mg
eGFR	n = 148	n = 147	n = 140	n = 132
	–15.0^†^ (–19.1, –10.8)	–5.4 (–9.6, –1.2)	–2.7 (–7.7, 2.4)	–3.4 (–8.5, 1.7)
SCr	n = 148	n = 147	n = 140	n = 132
	17.3^†^ (13.1, 21.5)	6.9 (2.6, 11.1)	5.4 (1.3, 9.5)	4.9 (0.7, 9.0)
Cystatin-C	n = 148	n = 147	n = 140	n = 130
	16.1^‡^ (11.8, 20.4)	11.4 (7.1, 15.8)	12.8 (8.7, 16.9)	14.8 (10.6, 19.1)
**RAAS markers**				
Renin*	n = 138	n = 138	n = 135	n = 127
	19.9 (–1.8, 46.3)	31.0 (7.6, 59.5)	12.9 (–7.4, 37.7)	14.6 (–5.9, 39.6)
Aldosterone*	n = 125	n = 122	n = 122	n = 114
	–10.8 (–21.8, 1.7)	–11.2 (–22.1, 1.2)	–5.5 (–16.6, 7.1)	2.1 (–9.8, 15.6)
Sodium excretion (fractional)*	n = 143	n = 139	n = 139	n = 130
	27.1^‡^ (10.8, 45.9)	4.6 (–9.0, 20.3)	0.9 (–12.1, 15.8)	–6.2 (–18.5, 8.0)
**Renal tubular markers**				
α-GST*	n = 96	n = 96	n = 84	n = 84
	12.7 (0.7, 26.2)	1.0 (–10.0, 13.3)	3.9 (–7.6, 16.9)	6.9 (–5.1, 20.4)
NAG*	n = 142	n = 140	n = 138	n = 132
	12.4 (–7.7, 37.0)	0.2 (–17.8, 22.3)	1.7 (–14.4, 20.9)	6.8 (–10.3, 27.2)

Data are from the full analysis population. The last-observation-carried-forward principle was applied to missing values of continuous variables.

α-GST = α-glutathione S-transferase; CI = confidence interval; eGFR = estimated glomerular filtration rate; EOF = end of follow-up; EOT = end of treatment; LS = least squares; NAG = N-acetyl-β-D-glucosaminidase; RAAS = renin–angiotensin–aldosterone system; SCr = serum creatinine.

*Analysis on log-transformed scale, geometric means ratio expressed as percentage change.

^†^p < 0.001 versus pioglitazone at EOT. ^‡^p < 0.05 versus pioglitazone at EOT.

SCr showed an inverse pattern of change to eGFR at the end of treatment and the end of follow-up. Increase in mean cystatin-C occurred in both treatment groups in parallel, with a greater increase in the aleglitazar group at the end of treatment, but remained elevated at the end of follow-up for both the aleglitazar and pioglitazone groups. Among other renal parameters investigated, no differences were observed (p > 0.05) with the exception of sodium excretion at the end of treatment, where change from baseline was greater with aleglitazar (p = 0.008).

**Figure 2:**
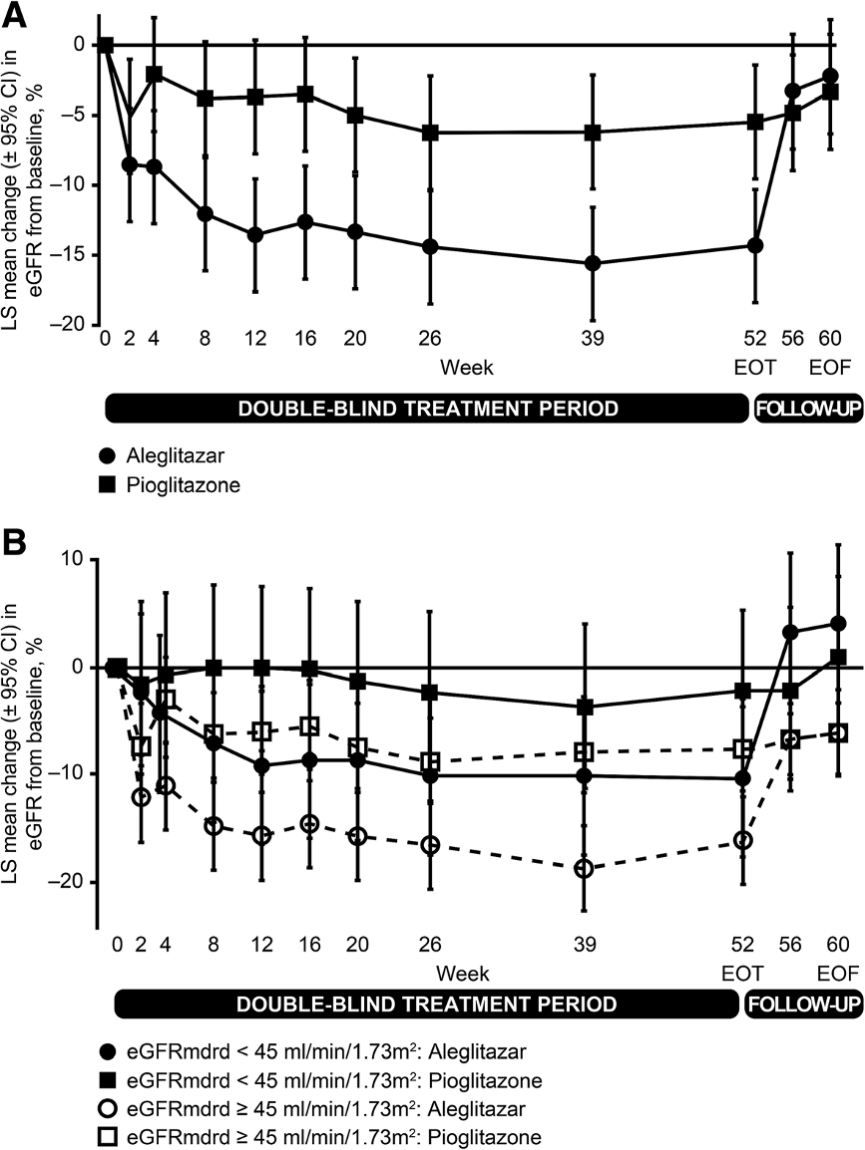
**LS mean percentage change in eGFR. (A)** Change from baseline over time (full analysis population). Last-observation-carried-forward principle was applied to missing values of continuous variables. eGFR = estimated glomerular filtration rate; LS = least squares; EOT = end of treatment; EOF = end of follow-up. **(B)** Change from baseline over time, by eGFR strata (full analysis population). Last-observation-carried-forward principle was applied to missing values of continuous variables.

**Figure 3:**
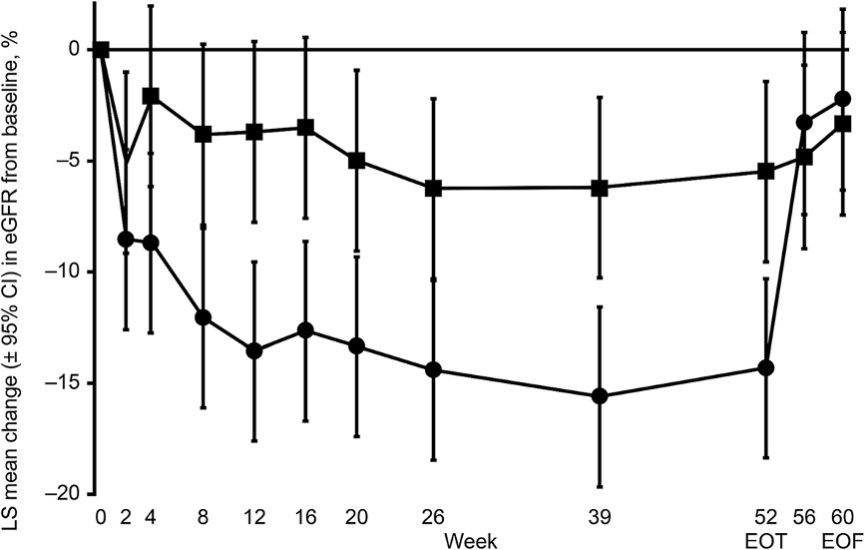
**LS mean percentage change in eGFR from baseline at end of follow-up by analysis population.** Circles = aleglitazar; squares = pioglitazone. LS mean change from baseline and ± 95% CI. Analysis of covariance of percentage change from baseline. Missing data imputed using last-observation-carried-forward principle applied only to follow-up measurements of serum creatinine ≥ 21 days after last treatment intake. CI = confidence interval; eGFR = estimated glomerular filtration rate; EOF = end of follow-up; EOT = end of treatment; LS = least squares. *Numbers reflect patients included in the full analysis population who had at least one follow-up measurement of serum creatinine ≥ 21 days after last treatment intake.

Reduction in mean eGFR from baseline was evident in the aleglitazar group by Week 2 and plateaued after 8 weeks, returning towards baseline following cessation of treatment (Figure 2A). Mean eGFR change at end of treatment with aleglitazar was –15.0% (95% CI: –19.1, –10.8) versus –5.4% (95% CI: –9.6, –1.2) with pioglitazone (p < 0.001). The treatment difference in eGFR at end of follow-up (the primary endpoint) was 0.77% (95% CI: –4.5, 6.0, p = 0.77), with lower 95% CI above –7.5%, thus establishing non-inferiority of aleglitazar versus pioglitazone. The same pattern of changes was observed in both eGFR strata (eGFR < 45 or ≥ 45 mL/min/1.73 m^2^) (Figure 2B). Absolute changes in eGFR from baseline to the end of follow-up were also similar in aleglitazar versus pioglitazone groups irrespective of eGFR strata (Table 3). The primary endpoint was met, irrespective of the analysis population studied (Figure 3).

**Table 3:** **Change in renal function from baseline in the overall cohort and by eGFR strata**

Cohorts	Aleglitazar 150 μg			Pioglitazone 45 mg		
	Mean baseline eGFR	EOT absolute change (95% CI)	EOF absolute change (95% CI)	Mean baseline eGFR	EOT absolute change (95% CI)	EOF absolute change (95% CI)
Overall cohort	n = 149	n = 148	n = 140	n = 148	n = 147	n = 132
	46.5	–7.3 (–8.9, –5.6)	–1.6 (–3.6, 0.4)	46.7	–2.8 (–4.5, –1.2)	–1.7 (–3.7, 0.3)
eGFR < 45 mL/min/1.73 m^2^	n = 69	n = 68	n = 63	n = 59	n = 58	n = 52
	37.7	–4.5 (–7.0, –2.0)	0.3 (–2.8, 3.4)	36.8	–0.8 (–3.4, 1.7)	–0.3 (–3.5, 2.8)
eGFR ≥ 45 mL/min/1.73 m^2^	n = 80	n = 80	n = 77	n = 89	n = 89	n = 80
	54.2	–9.2 (–11.5, –6.9)	–2.9 (–5.4, –0.2)	53.3	–4.3 (–6.5, –2.0)	–2.5 (–5.1, 0.1)

Data show LS mean absolute changes to EOT and EOF observed in the full analysis population. The last-observation-carried-forward principle was applied to missing values of continuous variables. CI = confidence interval; LS = least squares; EOT = end of treatment; EOF = end of follow-up.

#### UACR

**Figure 4:**
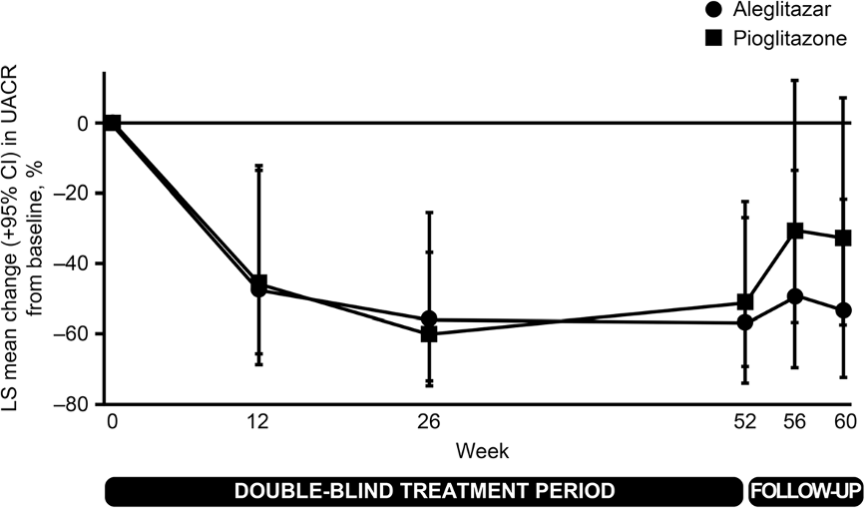
**LS mean percentage change in UACR from baseline over time (patients with macroalbuminuria at baseline).** Median baseline UACR values (interquartile range) were 75.4 mg/mmol (55.7–133.8) for aleglitazar (n = 21) and 89.6 mg/mmol (43.3–116.0) for pioglitazone (n = 27). Analysis on log-transformed scale, geometric means ratio expressed as percentage change. Patients analyzed at end of treatment (using last-observation-carried-forward principle): n = 20 for aleglitazar and 26 for pioglitazone, and at end of follow-up: n = 19 for aleglitazar and 25 for pioglitazone. LS = least squares; UACR = urine albumin-to-creatinine ratio.

In the full analysis population, reductions in UACR of 35.0% with aleglitazar (95% CI: –46.8, –20.5) and 29.4% with pioglitazone (95% CI: –42.4, –13.4) were observed between baseline and end of treatment, and of 19.8% with aleglitazar (95% CI: –36.3, 0.9) and 18.2% with pioglitazone (95% CI: –35.3, 3.3) between baseline and end of follow-up. In patients with macroalbuminuria at baseline (n = 48; > 80% of whom were on angiotensin-converting enzyme inhibitors [ACEis] and/or angiotensin receptor blockers [ARBs]), reductions in UACR of 59.3% with aleglitazar (95% CI: –76.6, –29.1) and 50.5% with pioglitazone (95% CI: –69.5, –19.5) were observed between baseline and end of treatment (Figure 4). The effect of aleglitazar was maintained to end of follow-up, with 54.1% (95% CI: –73.6, –20.2) reduction in UACR compared with 32.8% (95% CI: –58.5, 8.7) with pioglitazone.

#### Micro- and macroalbuminuria

At baseline, the majority of patients were categorized to normoalbuminuria based on UACR (91/149 [61%] aleglitazar versus 90/148 [61%] pioglitazone), followed by microalbuminuria (37/149 [25%] aleglitazar versus 31/148 [21%] pioglitazone). Patients with macroalbuminuria at baseline were 21/149 (14%) on aleglitazar versus 27/148 (18%) on pioglitazone. At the end of treatment, the following shifts in categories from macroalbuminuria at baseline were observed with aleglitazar and pioglitazone, respectively: one patient (0.7%) and two patients (1.4%) from macro- to normoalbuminuria; 11 patients (7.4%) and seven patients (4.7%) from macro- to microalbuminuria; eight patients (5.4%) and 17 patients (11.5%) remained macroalbuminuremic. In both treatment groups, for one patient (0.7%) with macroalbuminuria at baseline, end-of-treatment status for macroalbuminuria was not known.

#### Composite renal endpoints

One patient on aleglitazar and two patients on pioglitazone experienced the double composite renal endpoint event, while four patients in each group reached the triple composite renal endpoint event. No clinically relevant differences were observed between the groups regarding the composite renal endpoints. No patients in either group required acute dialysis, and one patient on pioglitazone required chronic dialysis.

### Lipid and glycemic parameters, body weight and blood pressure

Superior improvements in lipids and similar changes in glycemic control, body weight and blood pressure were observed with aleglitazar versus pioglitazone at end of treatment (Table 4).

**Table 4:** **Change from baseline to end of treatment: lipids, glycemic parameters, body weight and blood pressure**

Full analysis population; percentage change from baseline (95% CI)	Aleglitazar 150 μg	Pioglitazone 45 mg
LDL-C*	n = 142	n = 141
	–7.3^†^ (–13.2, –1.0)	–0.3 (–6.8, 6.6)
HDL-C	n = 142	n = 140
	22.0^‡^ (17.4, 26.6)	11.6 (6.9, 16.3)
Triglycerides	n = 142	n = 140
	–33.6^‡^ (–41.1, –26.1)	–14.1 (–21.7, –6.5)
**Safety analysis population; absolute change from baseline (95% CI)**	**Aleglitazar 150 μg**	**Pioglitazone 45 mg**
HbA1c, %	n = 148	n = 147
	–0.67 (–0.87, –0.48)	–0.76 (–0.96, –0.56)
FPG, mmol/L	n = 148	n = 147
	–1.96 (–2.38, –1.54)	–1.64 (–2.06, –1.22)
Body weight, kg	n = 149	n = 147
	2.4 (1.6, 3.2)	2.5 (1.7, 3.3)
Systolic blood pressure, mmHg	n = 149	n = 147
	1.7 (–1.0, 4.5)	2.3 (–0.4, 5.1)
Diastolic blood pressure, mmHg	n = 149	n = 147
	–2.2 (–3.9, –0.5)	–0.5 (–2.3, 1.2)

Data show LS mean changes. The last-observation-carried-forward principle was applied to missing values of continuous variables.

CI = confidence interval; FPG = fasting plasma glucose; HbA1c = glycosylated hemoglobin; HDL-C = high-density lipoprotein cholesterol; LDL-C = low-density lipoprotein cholesterol; LS = least squares.

*Analysis on log-transformed scale, geometric means ratio expressed as percentage change.

^†^p < 0.05 versus pioglitazone at end of treatment. ^‡^p < 0.001 versus pioglitazone at end of treatment.

### General safety and tolerability

Adverse events were reported in 67% of patients on aleglitazar compared with 68% on pioglitazone. No major safety concerns were identified. Selected adverse events and adjudicated CV events are summarized in Tables 5 and 6, respectively. Congestive heart failure was reported in five patients treated with aleglitazar. Four patients experienced at least one heart failure event positively adjudicated by the CEC, two of which were reported as serious adverse events and required hospitalization. Three patients treated with pioglitazone experienced at least one heart failure event, but none of these events were positively adjudicated by CEC.

**Table 5:** **Selected adverse events (safety analysis population)**

Patients with at least one AE, n (%)	Aleglitazar 150 μg (n = 149)	Pioglitazone 45 mg (n = 152)
Peripheral edema	18 (12)	30 (20)
Heart failure (investigator-reported)	5 (3)	3 (2)
Fractures*	3 (2)	2 (1)
Renal AEs	8 (5)	6 (4)
Muscular AEs	4 (3)	5 (3)
Hepatic AEs	2 (1)	3 (2)
Hypoglycemia^†^	29 (20)	22 (15)
Severe hypoglycemia	2 (1)	0
Malignancy^‡^	3 (2)	1 (1)

AE = adverse event.

*The fracture sites were ankle, foot and humerus for the aleglitazar cases and clavicle, rib and scapula for the pioglitazone cases (one of the patients in the pioglitazone group had two fractures).

^†^In both groups, approximately 90% of hypoglycemic events occurred in patients on sulfonylureas. The total number of hypoglycemic events was 100 in the aleglitazar group and 56 in the pioglitazone group.

^‡^Malignancies were not considered to be related to study medication, including one case of bladder cancer in the aleglitazar group with first symptoms reported 3 weeks after start of treatment. The other cases in the aleglitazar group related to breast cancer and metastases to neck, and in the pioglitazone group to squamous cell carcinoma of skin.

**Table 6:** **Adjudicated cardiovascular events and non-cardiovascular death (safety analysis population)**

Patients with at least one event, n	Aleglitazar 150 μg (n = 149)	Pioglitazone 45 mg (n = 152)
**MACE**		
Cardiovascular death	2	2*
Non-fatal myocardial infarction	0	1
Non-fatal stroke	0	1*
Non-cardiovascular death	1	1
**Congestive heart failure**		
Non-fatal heart failure (hospitalized)	2	0
Non-fatal heart failure (non-hospitalized)	2	0

*Non-fatal stroke and cardiovascular death occurred in the same patient, thus total number of patients experiencing at least one MACE event was two in aleglitazar group and three in pioglitazone group. The same patient has also experienced a myocardial infarction, as judged by the investigator (not included in the above summary), but insufficient information was provided to the Clinical Events Committee to conclusively adjudicate this event as myocardial infarction or unstable angina (cardiac enzymes data was not available). MACE = major adverse cardiovascular events.

## Discussion

The AleNephro trial evaluated the reversibility of effects of 52 weeks of treatment with aleglitazar as compared with pioglitazone on renal parameters in patients with T2D and moderate renal impairment (stage 3 CKD). As expected for a PPAR-α agonist [17–19], aleglitazar was associated with a reduction in eGFR during therapy that was not progressive, plateauing after 8 weeks, and was significantly greater than that of pioglitazone at end of treatment. The principal finding of this study was that the effects of both agents on renal function were reversible, with a decrease in eGFR with aleglitazar that was comparable (non-inferior) to that with pioglitazone by 8 weeks following withdrawal of treatment. Primary endpoint results were consistent using either the MDRD or the Cockcroft–Gault equation for eGFR calculation (data not shown). Furthermore, in patients with stage 3 CKD, MDRD provides an approximately equivalent estimation of eGFR compared with the CKD-Epi equation [20]. In addition to meeting the primary endpoint, AleNephro showed that the decrease in eGFR observed with aleglitazar during the treatment period reversed during follow-up in patients with eGFR < 45 mL/min/1.73 m^2^. Hence, even in this high-risk group, aleglitazar did not adversely affect renal safety, similar to pioglitazone and fenofibrate [14, 21]. These reversible changes in eGFR and SCr are consistent with previous observations from SESTA-R [10] and confirm data from the AleCardio trial, which investigated the effect of aleglitazar on cardiovascular outcomes after acute coronary syndrome [12].

Although the mechanism of aleglitazar’s effect on eGFR and SCr is unclear, the reversibility of the effect resembles that of PPAR-α class of compounds, the fibrates, and inhibitors of the RAAS system, on these renal function markers, which paradoxically leads to renoprotection rather than acute kidney toxicity [18, 19, 22]. For example, in the Fenofibrate Intervention and Event Lowering in Diabetes (FIELD) trial, the increase in SCr during fenofibrate therapy returned to baseline following treatment cessation and was accompanied by significantly less decline in renal function (as measured by eGFR) compared with placebo [19]. A meta-analysis comparing the effects of ACEi/ARB with other antihypertensives demonstrated a consistent renoprotective effect of ACEi/ARB over other antihypertensive drugs (and placebo) in patients with T2D, independent of blood-pressure-lowering effects [22].

The mechanism for eGFR reduction with PPAR-α agonists is not well characterized. It may involve pre- or post-glomerular effects, including hemodynamic effects, or a combination of different mechanisms. Preclinical studies have revealed that PPAR-α agonists increase urinary fractional sodium excretion by decreasing Na^+^-K^+^ATPase activity in the proximal tubule [23] and altering cumulative sodium balance [24]. This is consistent with the increase in urinary fractional sodium excretion seen during aleglitazar treatment in AleNephro, which resolves during follow-up. Based on the effect of PPAR-α on proximal tubular sodium handling, one working hypothesis for the reduction of GFR observed in clinical studies may be tubuloglomerular feedback-induced hemodynamic changes. Elevated single-nephron GFR is an unfavorable glomerular hemodynamic compensatory mechanism for progressive nephron loss in patients with diabetic nephropathy [25]. Thus, lowering of GFR by hemodynamic effects leading to lower capillary pressure, which has been hypothesized for PPAR-α activation, may be protective.

In studies of ACEis and ARBs (e.g. RENAAL and ONTARGET), an initial decline in eGFR is often observed, most likely due to hemodynamic changes [26]. However, rather than being detrimental, this appears to correlate with renal protection in the long term [22, 26–28]. For example, in the RENAAL trial, losartan reduced UACR by 35%, associated with an 18% reduction in the rate of decline of renal function and 28% reduction in the risk of ESRD [27]. Assuming that the physiology associated with the initial drop in eGFR is similar across these different therapies, it may be speculated that AleNephro data indicate possible renoprotection, with reductions in UACR of 30–35% observed with aleglitazar and pioglitazone at treatment end, similar to previous UACR/proteinuria findings for pioglitazone [29, 30]. In patients with baseline macroalbuminuria, > 80% of whom were already on stable doses of ACEis and/or ARBs, UACR decreased from baseline to treatment end by 59% with aleglitazar and 50% with pioglitazone. The effect of aleglitazar on UACR in the range of proteinuria or very high albuminuria was maintained after drug discontinuation to end of follow-up. Clear evidence could only come from a large clinical outcomes study with hard clinical endpoints, which was beyond the scope of this renal function study.

At the end of treatment, the renal marker cystatin-C was elevated with aleglitazar relative to pioglitazone, although the levels were comparable by end of follow-up. This pattern in cystatin-C levels with aleglitazar is consistent with the ACCORD renal study [31] and the FIELD Helsinki substudy [32], which also showed an initial increase in cystatin-C during fenofibrate treatment and reversibility following treatment discontinuation. A contributing factor to elevations in cystatin-C may be the anti-inflammatory effects of aleglitazar and pioglitazone, which can result in upregulation of cystatin-C synthesis [33], but hemodynamic effects (as hypothesized for SCr) may also drive cystatin-C changes. In terms of the RAAS markers, in both treatment groups, renin levels remained elevated at end of follow-up, while aldosterone was slightly reduced, although these changes were not significant between treatment groups. As in SESTA-R [10], elevations in the renal tubular markers α-GST and NAG were observed with both aleglitazar and pioglitazone; however, treatment differences were not considered clinically significant.

No major safety concerns were identified with aleglitazar in AleNephro, and no increase in adverse events was noted in patients with reduced eGFR compared with previous studies. The incidence of edema was numerically lower in patients treated with aleglitazar versus pioglitazone, although the incidence of investigator-reported congestive heart failure events was higher in patients treated with aleglitazar. However, the total number of adjudicated CV events was similar between groups. The imbalance in hypoglycemia events between the groups appears to be largely driven by a subset of seven patients with multiple (7–12) reported events accounting for 65 out of 100 events (versus three patients with six to eight events each in the pioglitazone group accounting for 21 out of 56 events). In both groups, 90% of hypoglycemic events occurred in patients on sulfonylureas.

The incidence of bone fractures was similar for aleglitazar and pioglitazone and none of the malignancy cases were considered by the investigators to be drug related. For the bladder cancer case in the aleglitazar group, first symptoms were reported 3 weeks after treatment commenced, suggesting it was pre-existing and unlikely to be treatment related, but detected due to the protocol-imposed monitoring for this cancer.

Acknowledging possible deficiencies of the last-observation-carried-forward method, the robustness of the primary analysis was checked by performing two sensitivity analyses using the per-protocol and completers populations. Both confirmed the conclusions of the primary analysis (data not shown). Only a small proportion of observations available for the primary analysis was missing (8.5%), and was therefore unlikely to have a large impact on the interpretation of the results. Survival type analyses were not performed for the primary endpoint as they were not foreseen by the protocol. Also, the interpretation of such analyses may be limited due to a potentially small number of events.

AleNephro confirmed glycemia and lipid findings from SYNCHRONY [9]. Aleglitazar (a balanced PPAR-α/γ activator) was found to be similar to the PPAR-γ agonist pioglitazone with respect to glycemic control (HbA1c and FPG), but, presumably due to its activity on PPAR-α, superior for control of atherogenic dyslipidemia.

## Conclusions

In conclusion, AleNephro data indicated that aleglitazar-induced reduction in eGFR plateaued after 8 weeks and was significantly greater than that of pioglitazone at end of 52 weeks’ treatment. Following treatment withdrawal, the decrease in eGFR caused by aleglitazar reversed and eGFR values were comparable (non-inferior) to that with pioglitazone. Aleglitazar was generally well-tolerated, with similar safety signals as pioglitazone treatment, although we acknowledge that the small size of the study is a major limitation regarding safety assessment. Even though aleglitazar’s development program was halted due to lack of efficacy in CV outcomes indicating no CV benefit and PPAR-related class side effects in the post-ACS population following a recent regular safety review of the AleCardio Phase III trial [12], we believe that our findings provide important insights into the renal effects of dual PPAR-α/γ activation.

## Electronic supplementary material

Additional file 1: Table S1: Full list of ethics committees and independent review boards. (DOCX 40 KB)
