# Supplementary material for: Effects of the dual peroxisome proliferator-activated receptor-α/γ agonist aleglitazar on renal function in patients with stage 3 chronic kidney disease and type 2 diabetes: a Phase IIb, randomized study
Source: BMC Nephrol. 2014 Nov 18;15:180. doi: 10.1186/1471-2369-15-180 (PMC4364102; doi:10.1186/1471-2369-15-180)
Supplement: Supplementary file 1 — Additional file 1: Table S1: Full list of ethics committees and independent review boards. (DOCX 40 KB) [file 12882_2013_895_MOESM1_ESM.docx]

**Supplementary Table 1: full list of ethics committees and independent review boards**

| **Country** | **Ethics committee/independent review board** |
| --- | --- |
| AUSTRALIA | Human Research Ethics  Committee (TQEH/LMH/MH), 28  Woodville Road Woodville South SA 5011, Australia |
|  | SSWAHS Ethics Review  Committee (RPAH Zone), c/- Research Development Office Level 3, Building 92, Missenden Road,  2050, Camperdown, New South Wales, Australia |
|  | The Alfred Hospital  Research Ethics Committee, The Alfred Hospital,  55 Commercial  Road, 3004, Melbourne,  Victoria, Australia |
|  | HREC (Tasmania)  Network, Office of Research Services University of Tasmania,  Private Bag 01, 7001,  Hobart, Tasmania |
| BRAZIL | Comitê de Ética em  Pesquisa do Hospital Moinhos de Vento,  Rua Ramiro Barcelos, 910 -  Bloco D, Brazil |
|  | CEP da Irmandade da  Santa Casa de Misericórdia de São Paulo – ISCMSP |
|  | Comite de Etica em  Pesquisa da Universidade Federal do Ceara, Rua Coronel Nunes de Melo,  1127 – Rodolfo Teofilo,  Fortaleza – CE / 60430- 275, Brazil |
|  | CEP da Irmandade da  Santa Casa de Misericórdia de São Paulo – ISCMSP, Rua Santa Isabel, 305 - 4° Andar, Santa Cecília, 01221-010, São Paulo, SP, Brazil |
| COLOMBIA | Comité de Investigaciones y ética en Investigaciones,  Calle 78 B NO. 69-240,  Medelllin, Colombia |
|  | C. De Etica En Invest. De  La Clinica De La Costa, Carrera 50 # 80-90 Piso 2, Barranquilla, Colombia |
|  | Comité de Etica en la  Investigacion CAIMED, Carrera 42 A No. 17 - 50, Bogotá, Colombia |
| EL  SALVADOR | Comite Nacional de Etica de Investigación Clinica, Consejo Superior de Salud Pública  Paseo General Escalon,  No 3551  San Salvador, El Salvador |
| GERMANY | EK Cottbus LÄK Brandenburg,  Dreifertstr. 12,  03044 Cottbus,  Germany |
| HONG KONG | Joint CUHK-NTEC Clinical  Research Ethics Committee, Flat 3C, Block B, Staff Quarters, Prince of Wales Hospital, 852, Shatin, Hong Kong |
|  | NTW Cluster Clinical &  Research Ethics Committee, NTW Cluster Clinical & Research Ethics Committee, Hong Kong |
| HUNGARY | Medical Research Council,  Ethics Committee for Clinical Pharmacology, Budapest  Arany János u. 6.-8., H- 1051,  Hungary |
| ITALY | CE Centrale della  Fondazione Salvatore Maugeri, via Ferrata, 8, 27100, Pavia, Italy |
|  | Ethics Committee,  University Hospital San Martino,  largo Rosanna Benzi 10, 16132 Genova,  Italy |
|  | Ethics Committee of the  Foundation Raffaele Del Monte Tabor,  via Olgettina, 60,  20132 Milano,  Italy |
| MEXICO | Comité de Ética Hospital  CIMA Chihuahua, Hacienda del Valle # 7120,  Fraccionamiento las  Haciendas, 31238,  Chihuahua,  Mexico |
|  | Inst. Jalisciense De  Investigacion Clinica Sa, Penitenciaria # 20. Col. Centro, 44100,  Guadalajara,  Mexico |
|  | CB para la Investigación  Clínica Pachuca, José María Bandera No. 407-A  #6, Colonia Doctores,  42090, Pachuca,  Mexico |
|  | Inst. Jalisciense De  Investigacion Clinica Sa, Penitenciaria # 20. Col. Centro, 44100,  Guadalajara,  Mexico |
|  | ReMeDi, Bioethics  Committee for Clinical Investigations, Marian Arista No. 330, Office D, Col. Doctores. C.P. 42090,  Pachuca, Hgo,  Mexico |
| PERU | Comité Institucional de  Etica en Investigación de la Asociación Benéfica Prisma, Carlos Gonzales  #251 Urbanización Maranga San Miguel,  Lima 32,  Peru |
|  | Comité de Etica del Hospital Rebagliati,  Av. Rebagliati 490,  Jesus Maria,  Lima 11,  Peru |

| ROMANIA | Comisia Nationala de  Etica, Str. Anton Cehov, nr. 8, sect. 1,  Bucuresti,  Romania |
| --- | --- |
| RUSSIA | Local Ethics Committee,  Federal Agency for Healthcare and Social Development, K-67, 47,  Piskarevskiy pr., 195067 Saint-Petersburg, Russia |
|  | Ethics Committee, City  Hospital of Holy Martyr Elizabeth, Vavilovs Street, 14, 192257 St.  Petersburg, Russia |
|  | Ethics Committee, State  Institution of Healthcare, Yaroslavl Regional Clinical Hospital, 7 Yakovlevskaya  St., 150062 Yaroslavl,  Russia |
|  | Ethics Committee,  Academy I.P. Pavlov Saint-Petersburg State Medical University, 10,  Rentgena St., 197101  Saint-Petersburg,  Russia |
|  | Local Ethics Committee,  Chelyabinsk Municipal Institution of Healthcare, City Clinical Hospital No. 6, 454136 Chelyabinsk,  Russia |
|  | Interuniversity Ethics  Committee, 8,  Trubetskaya str., 119992 Moscow,  Russia |
|  | Ethics Committee, V.A.  Almazov Federal Heart, Blood and Endocrinology Center of Minzdravsocrazvitia Rossii, 15, Parkhomenko  Av., 194156 Saint- Petersburg,  Russia |
| SLOVAKIA | Etická Komisia  Samosprávneho Kraja, Komenského 48,  01109 Zilina,  Slovakia |
|  | Etická Komisia  Nitrianskeho Samosprávneho Kraja, Stefánikova Trieda 69,  94909 Nitra,  Slovakia |
|  | Ethics Committee  bratislavskeho samopravneho kraja, Sabinovska ul c 16, 820  05 Bratislava,  Slovakia |
